# Editorial: Patient Empowerment and Person-Centered Care in Movement Disorders

**DOI:** 10.3389/fneur.2020.00317

**Published:** 2020-04-28

**Authors:** Adolfo Ramirez-Zamora, Pedro Chana, Mayela Rodríguez-Violante

**Affiliations:** ^1^Department of Neurology, Norman Fixel Institute for Neurological Diseases, University of Florida Health, Gainesville, FL, United States; ^2^Central Disorders of the Movement, University of Santiago de Chile, Santiago, Chile; ^3^Departamento de Neurologia, Instituto Nacional de Neurología y Neurocirugía Manuel Velasco Suárez, Mexico, Mexico

**Keywords:** patient empowerment, person-centered, centeredness, shared decision making, Parkinsons disease, cerebral palsy, movement disorders

The traditional practice of medicine in recent years continues to evolve to incorporate the perspectives and goals of caregivers and patients in their care. Accordingly, there is an increased interest in additional research in this field, aimed to determine if new techniques and patient centered approaches correlate with better outcomes and quality of life. This is a constantly changing environment that transcends multiple disciplines, but it is particularly important in patients with neurodegenerative disorders. Concepts such as patient empowerment, self-efficacy, health literacy, shared decision-making, patient-centricity, or centeredness are becoming increasingly common in the medical literature.

A variety of topics are discussed in this special issue, ranging from discussions related to deep brain stimulation (DBS), sex differences in movement disorders, patients' communication, and perspective in healthcare, to the potential use of innovative technologies.

Joshi et al. report the results of a single-arm, open-labeled, observational study conducted at two US academic institutions to assess the utility of the Personal Kinetic Graph (PKG) System in routine clinical care of patients with Parkinson's disease (PD). In this study, physicians discussed PD symptoms with patients and conducted a motor examination prior to reviewing the PKG report and comparing it to their initial assessments. They concluded that the PKG system provided insights for treatment plans in 79% of patients. Physicians found improved patient dialogue in 59% of visits, along with improved ability to assess treatment impact and motor assessment in a third of visits.

Rastgardani et al. conducted a survey and questionnaires to assess the frequency, content, and ease of communication about OFF periods in PD. Good communication between patients and health care providers is critical to improve care and accurate communication of OFF periods in PD, as this has been particularly challenging. The authors administered an online cross-sectional survey to understand the opinions and experiences of patients and providers to better define barriers and facilitators of communication between them. The most common physician-reported barriers to communication were a patient's cognitive impairment, difficulties recognizing OFF times, and poor patient understanding of OFF periods' relationship to medication timing. The barriers most commonly noted by patients were that they perceived OFF periods to be part of the disease and not necessarily a change in symptoms that can be improved/managed with medical adjustments, variability of symptoms, and difficulty in describing symptoms.

Nijhuis et al. conducted a cross-sectional survey that explored patient involvement in medical decisions and identified facilitators and barriers for shared decision-making in advanced PD patients. Their goal was to identify barriers and to better understand decision making among different advanced therapies used for refractory motor fluctuations in PD including DBS, Levodopa-Carbidopa intestinal gel, or continuous subcutaneous Apomorphine infusion. In their sample, patients preferred to be part of the decision-making and most respondents engaged in an active role. In about half of patients, preferred role did not match experienced role and about 30% had a more active role than they would have preferred. Participants identified the most important facilitators for shared decision-making at the patient's level, at the neurologist's level, and within the professional-patient relationship. Also, their results highlight that education is critical for making decisions as often patients do not know all treatment options.

Vlaanderen et al. present their results of a study analyzing a national administrative medical claims database containing data of all newly diagnosed PD patients between 2012 and 2016 in the Netherlands. Specifically, they aimed to investigate sex differences among patients early in the disease. Secondary analysis included information about relevant complications and clinical milestones, namely, unexpected hospitalizations, pneumonia, orthopedic injuries, nursing home admission, and death. Using these data, they constructed a patient journey stratified for sex. Their study is important as sex differences in PD start to emerge. Their results suggest that women experienced more complications and they accessed more healthcare services then men (visits to general practitioners and physiotherapists) early in their disease.

Discussions and counseling patients about the long-term effects of neuromodulation are difficult due to a variety of factors ranging from disease heterogeneity, comorbidities, and disease progression. In their report, Zhou et al. present the results of investigating the acute long term effects of subthalamic nucleus (STN) DBS in 11 young onset PD patients followed at Ruijin Hospital, Shanghai, China for over a decade. Motor assessments were rated by a blinded neurologist not involved in the study. Patients showed a significant improvement in motor symptoms both in the off-medication and on-medication state with >50% reduction in the total UPDRS-III scores. Specifically, improvement in axial symptoms including gait was noted long term. However, the authors emphasized that postural stability did not improve at last follow up.

Hsu et al. conducted a meta-regression analysis of randomized clinical trials assessing the effect of therapeutic exercise intensity on cerebral palsy (CP) outcomes. Intensive physical therapy or exercise has been associated with favorable CP outcomes, but there are few studies investigating the unique effects of exercise intensity and duration. Using the Gross Motor Function Measurement (GMFM) as their main outcome in CP, they performed a Meta regression analysis of 13 trials that recruited 412 children with CP. Their analysis suggests that improvement in GMFM scores was positively associated with the number of daily training hours and program duration. Intensive physical exercise improved CP outcomes in the intervention and standard therapy groups. The duration of therapeutic intervention was important and correlated with better outcomes, while an increase in the number of daily training hours improved outcomes in the children who received standard therapy. Their study is the first relating intensity of common therapeutic exercises and gross motor function improvement. It appears that a daily dose of exercise is an important variable for improvements. Further studies are needed in more controlled and specific populations.

## Author Contributions

AR-Z participated in drafting the article. AR-Z, PC, and MR-V participated in the writing and revision of the manuscript.

## Conflict of Interest

The authors declare that the research was conducted in the absence of any commercial or financial relationships that could be construed as a potential conflict of interest.

